# Supplementary figures and images for: Comparative proteomics of HepG2 cells reveals NGLY1 as an important regulator of ferroptosis resistance and iron uptake
Source: PLoS One. 2025 Aug 14;20(8):e0328166. doi: 10.1371/journal.pone.0328166 (PMC12352660; doi:10.1371/journal.pone.0328166)

Supplemental Figure 1

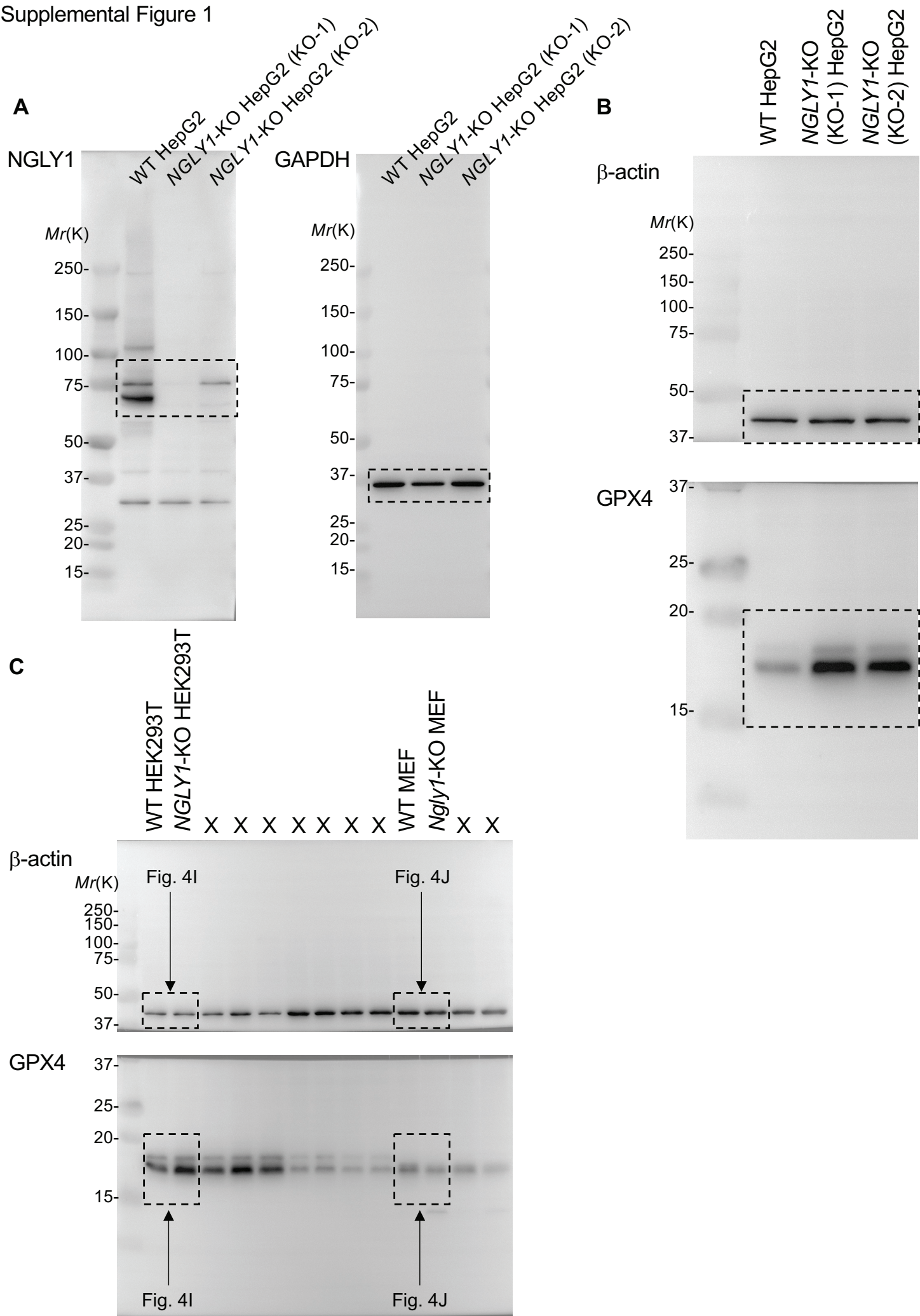

Supplement: S1 Fig — (PDF) [file pone.0328166.s001.pdf]
