# Supplementary material for: Comparative proteomics of HepG2 cells reveals NGLY1 as an important regulator of ferroptosis resistance and iron uptake
Source: PLoS One. 2025 Aug 14;20(8):e0328166. doi: 10.1371/journal.pone.0328166 (PMC12352660; doi:10.1371/journal.pone.0328166)
Supplement: Attachment — Submitted filename: Response-to-the-reviewers-final.docx [file pone.0328166.s004.docx]

**Response to the reviewers’ comments**

We wish to extend our profound gratitude to the reviewers for their constructive comments. In light of the reviewer’s comments, a number of supplementary experiments were conducted. During the course of these new experiments, we were not able to reproduce our originally reported decrease in GPX4 protein levels in *NGLY1*-KO HepG2 cells compared to WT HepG2 cells. In order to ensure the highest possible degree of reproducibility, the recent data set was reproduced independently by two researchers, yielding consistent results. This recent data set revealed no reduction in GPX4 protein levels in *NGLY1*-KO HepG2 cells under any of the tested experimental conditions. In contrast, this recent data set displayed an increase in the GPX4 protein levels in *NGLY1*-KO HepG2 cells in comparison with WT HepG2 cells, within the specified experimental parameters. These recent findings are more consistently aligned with the previously stated increase in GPX4 mRNA expression levels as outlined in the original submission.

Further experiments were conducted to assess the sensitivity of *NGLY1*-KO and WT HepG2 cells to ferroptosis induction by GPX4 inhibitors (RSL3 and ML162). *NGLY1*-KO HepG2 cells exhibited increased resistance to induced ferroptotic cell death in comparison to WT HepG2 cells. These findings are consistent with previous reports associating elevated GPX4 levels with increased resistance to induced ferroptotic cell death, thereby providing additional support for our present finding of increased GPX4 protein levels in *NGLY1*-KO HepG2 cells. In light of this aforementioned consistency of our present findings concerning elevated GPX4 protein levels in *NGLY1*-KO HepG2 cells, we have revised our manuscript to reflect our recent data set, as detailed below.

**Revisions to Figures**

**1.** Fig. 2: previous Figs. 2A-B were removed; Fig. 2C was moved to Fig. 3D; previous Figs. 3D-F were moved to Figs. 2G-I; new panels were added as Figs. 2E-F showing population mappings of flow cytometry

**2.** Fig. 3; as stated above, Figs. 3D-F were moved to Fig. 2G-I; previous Fig. 2C was moved to Fig. 3D; newly obtained GPX4 western blot results were added as Figs. 3E-F.

**3.** Fig. 5; previous Fig. 4 was moved to Fig. 5.

**4.** Fig. 4; new figures were added showing the present data set of ferroptosis sensitivity assays.

**Revisions to Supplemental Figures**

**1.** Fig. S2; Fig. S1 was moved to Fig. S2.

**2.** Fig. S1; new figures were added showing the present data set of ferroptosis sensitivity assays and western blotting of GPX4 using HEK293T and MEF cells.

*In accordance with the above outlined revisions, all associated Figure references in the text and associated Figure legends were revised.

**Revisions to** “**Results**” **section**

**1.** The new results were outlined in the second and third subsection of the ‘Results’ section. The titles of the two sections were revised from “***NGLY1 deletion had significant effects on GPX4 levels***” and “***NGLY1 deletion increased iron content in human liver cells***” to “***NGLY1 deletion has resulted in increased lipid peroxidation and ROS in HepG2 cells***” and “***NGLY1 deletion has significant effects on GPX4 levels and iron content***” (pages 10-12, lines 250-290).

**2.** A fourth subsection titled ‘***NGLY1 deletion significantly increased resistance against ferroptosis in HepG2 cells***’ was included to explain the new findings based on the ferroptosis induction experiments (pages 12, line 291-300). To better reflect these recent findings, the end of the last sentence of the first subsection of the ‘Results’ section (page 10, line 249) was changed from “impairing the inhibitory mechanism that suppress ferroptosis” to “affecting the inhibitory mechanism that suppress ferroptosis”.

**Revisions to “Discussion” section**

**1.** page 13, lines 311-312: To better reflect our recent findings we removed the sentence “Notwithstanding the observed upregulation of GPX4 mRNA, the levels of GPX4 protein were significantly reduced in *NGLY1*-KO HepG2 cells, indicating that GPX4 is subjected to post-translational down-regulations.”. And changed the beginning of the following sentence from “Fluorescence microscopy~” to “Additionally, fluorescence microscopy~”.

**2.** page 13, lines 313-315: The following sentence was inserted “The observed increase in GPX4 mRNA transcription in *NGLY1*-KO (KO-1) HepG2 cells, concomitant with increased GPX4 protein levels in *NGLY1*-KO HepG2 cells, was observed (Figs. 3D-F).”.

**3.** page 13, line 315: The start of the sentence was changed from “These findings suggest~” to “Together, these findings suggest~”.

**4.** page 13, lines 317-323: The final sub-sentence of the last line in the first paragraph of the ‘Discussion’ section was changed from “following NGLY1 deletion, offering a novel perspective on the molecular mechanisms underlying iron-related oxidative stress.” to “, while concomitantly increasing GPX4 expression as a remedial measure. *NGLY1*-KO HepG2 cells showed a higher resistance to induced ferroptotic cell death compared to WT HepG2 cells, though clonal differences regarding the extent of resistance were observed (**Fig. 4**). Both ferroptosis inducers utilized in this study, RSL3 and ML162, have been shown to target GPX4. However, the elevated expression of GPX4 in *NGLY1*-KO HepG2 cells results in an increased resistance to the induced ferroptotic cell death. These findings further support the notion that elevated GPX4 expression exerts a protective function in counteracting persistent ferroptotic stress in *NGLY1*-KO HepG2 cells”.

**5.** page 14, lines 347-349: The text passage “However, GPX4 protein levels exhibited a significant decline in *NGLY1*-KO (both KO-1 and KO-2) cells **(Figs. 2A-B)**, thereby constraining their capacity to neutralize ROS-driven lipid peroxidation. It remains to be determined whether the GPX4 protein downregulation in *NGLY1*-KO HepG2 cells is the result of a reduction in protein translation, disruption of post-translational modification or reduced protein stability” was revised to “Concomitantly, GPX4 protein levels exhibited significant increases in *NGLY1*-KO HepG2 cells (Figs. 3E-F), likely enhancing the cells ability to endure the consequences of elevated ROS-driven lipid peroxidation (Figs. 2A, 2C, 2E and 2I).”.

**6.** page 14, lines 355-358: The following text passage was added: “In the presence of ferroptosis inducers RSL3 and ML162 (32, 33), WT HepG2 cells exhibited significantly higher sensitivity towards both compounds in comparison to *NGLY1*-KO HepG2 cells (Figs. 4A-D). This suggests that *NGLY1*-KO HepG2 cells may exist in a partially adapted, ferroptosis-tolerant state which has been bolstered by increased GPX4 levels (Figs. 3E-F).”

**7.** pages 14-15, lines 361-371: An additional paragraph discussing the new findings was inserted, stating: “Earlier reports indicated the heightened sensitivity to ferroptotic cell death of *NGLY1*-KO A549 cells as well as their diminished GPX4 protein levels (7). The public database “NGLY1 browser (https://apps.embl.de/ngly1browser/)” reports that NGLY1 deficiency patient-derived fibroblasts show increased GPX4 mRNA and reduced GPX4 protein levels, while patient-derived lymphoblastoid cells showed decreased GPX4 mRNA and protein levels. Moreover, preliminary results indicate that *NGLY1*-KO HEK293T cells exhibit increased resistance to induced ferroptotic cell death, accompanied by elevated GPX4 protein levels when compared with WT HEK293T cells. Conversely, *Ngly1*-KO MEF cells exhibited heightened sensitivity to induced ferroptotic cell death, characterized by reduced GPX4 protein levels in comparison to WT MEF cells (**Figs. S1A-F**). Taken together, these results highlight the context dependency of induced ferroptotic cell death resistance and GPX4 mRNA and protein expression levels on *NGLY1*-KO cell type.”.

**8.** page 15, line 382: “GPX4 mRNA level” was changed to “GPX4 level”.

**9.** page 16, line 389: A sentence was added, and the beginning of the following sentence was adjusted to improve flow as follows: “Such a regulated or partial ferroptotic~” changed to “Alternatively, the enhanced expression of ferroptosis-resistance markers such as GPX4 may raise the threshold required to trigger ferroptotic cell death. This regulated or partial ferroptotic~”. In the same sentence the words “stress adaption” were changed to “stress adaptation”.

**Revisions to “Methods” section**

1. The method for “Ferroptosis inducer treatment of HepG2 cells” was added to the methods section (page 9, lines 207-212).

In the following section a point-by-point response to the reviewers’ comments is provided.

**Reviewer #1:**

Comments:

In this manuscript, Emmerson et al. report that cytosolic N-glycanase (NGLY1) is a regulator of iron homeostasis and ferroptosis in hepatoma cells. The authors found that the expression levels of several key factors for ion metabolism and related oxidative stress in NGLY1 KO HepG2 cells. Concomitantly, they demonstrated that the KO cells display the increased levels of ferrous, ROS and peroxidized lipids. Overall, although the mechanistic investigation was not fully performed, the conclusions of the paper are clear and all the methods are scientifically sound. I am happy to support publication of the paper after appropriate revision. Specific points are as follows.

Major points
1. The mechanism of how GPX4 protein is downregulated is not examined. To explain this phenomenon in NGLY1 KO cells, any logical hypothesis or experimental evidence should be provided.
As outlined above in more detail, two independent researchers confirmed the upregulation of GPX4 protein levels in *NGLY1*-KO HepG2 cells. This highly reproducible increase in GPX4 protein levels is now consistent with the reported increase in GPX4 mRNA levels. The new experimental results are shown in Figs. 3E-F.

2. The importance of the alterations of the key factors shown should be provided more. For instance, rescue experiments by exogenous expression of GPX4 or another key factor downregulated in NGLY1 KO would be one choice.

To assess this point, we treated the cells with GPX4 inhibitors (RSL3 and ML162) and checked their sensitivity to inhibitor induced ferroptotic cell death. The results are shown in new Figs. 4A-D. The high GPX4 expression resulted in a high resistance to the induced ferroptotic cell death. This indicates that the alteration of GPX4 levels in *NGLY1*-KO HepG2 cells plays an important role in the acquisition of ferroptotic cell death resistance compared to WT HepG2 cells. The same relationship between differential GPX4 expression level in *NGLY1*-KO cells and resistance to ferroptotic cell death was also observed in other cell lines (HEK293T and MEF cells, Fig. S1).

3. Cell type specificity is not discussed or examined. Analysis of previous or public proteomic or transcriptomic data from systemic KO mice or NGLY1-deficient patient-derived cells could provide a new clue.

Taking the reviewers suggestion into consideration, we checked the “NGLY1 browser” (<https://apps.embl.de/ngly1browser/>) public database. GPX4 mRNA and protein expression levels in NGLY1 deficiency patients-derived fibroblasts and lymphoblastoid cells are reported. Interestingly, the expression levels of GPX4 varied between these cell types at both the mRNA and protein levels. In addition, we performed further experiments using HEK293T and MEF cells to further probe cell type specificity. As a result, *NGLY1*-KO and WT HEK293T cells showed similar levels of sensitivity to ferroptosis induction and GPX4 expression as HepG2 cells. However, *Ngly1*-KO and WT MEF cells showed the opposite result. The results from HEK293T and MEF cells are shown in Supplemental Figs. 1A-F.

4. Fig. 2G. The authors showed the increase in neutral lipids in KO cells. Please provide more detailed and reasonable explanation of the Fig. 2F and G data.

We have added flow cytometry population mapping data (Figs. 2E-F), which shows an increased proportion of lipid peroxide-positive cells within the lipid-positive population in *NGLY1*-KO (KO-1) compared to WT HepG2 cells. This suggests that the elevated lipid peroxidation observed in *NGLY1*-KO HepG2 cells is not merely a consequence of increased lipid content. We have included this explanation in the main text (page 11, lines 260-264).

Minor points
5. Line 30-31. Structure of this sentence needs to be revised. “was” meant to be “were”?

Taking the reviewers comment carefully into consideration, we rechecked the sentences grammar. We are of the opinion that the subject the verb in question (“was”) refers to is “ferroptosis”. We remain of the believe that “was” is the grammatically correct verb and thus have chosen not to change the sentence in question (page 2, line 31).

6. Methods. Regarding centrifugation conditions, only “rpm” but not “g” is written in some places. Please define with g or rpm with the rotor name(s).
Following the reviewer’s suggestion, we changed the description from “rpm” to “*g*”. (pages 8-9, lines 180, 188, 206)

7. L216. “sequence confirmation” needs to be revised with more detailed description. For instance, genomic sequencing.

In accordance with the reviewer’s recommendation, we have changed the description from “sequence confirmation” to “genomic sequencing”. (page 9, line 222).

8. Fig. 2C. The aspect ratio of the graph seems strange and should be corrected.

The aspect ratio of the graph in question has been corrected and the figure has been moved to Fig. 3D in the revised manuscript.

9. Typo
Line 102. Space is needed between value and unit, “1.0g/L”.
The manuscript was revised as suggested (page 5, line 102).

Line 121. NP-40 should be spelled out when it first appears.
The manuscript was revised as suggested (page 5, line 121).

Line 123. Space is needed for “pH8.0”.
The manuscript was revised accordingly (page 5, line 123).

Line 257. Space is missing for 595nm and 520nm.
The manuscript was revised as suggested (page 11, line 255).

Line 333. “are” should be “is”?

The manuscript was revised as suggested (page 15, line 375).

**Reviewer #2:**

Comments:

The authors present an analysis of the consequences of loss of N-glycanase (encoded by the gene NGLY1) on cellular metabolism, with a particular focus on pathways implicated in oxidative stress and iron metabolism, using a human hepatocellular carcinoma cell line HepG2 cells. Loss of NGLY1 function is observed in the rare human genetic disorder NGLY1 Deficiency, characterized by global developmental delay, a hyperkinetic movement disorder, transient elevation of liver transaminases, peripheral neuropathy, and other features. The goal of this study is to probe the molecular mechanisms by which loss of N-glycanase drives these diverse features.

The manuscript is clear and presents some novel findings, notably reporting on disturbances in iron metabolism in NGLY1 deficient cells. However, my only real criticism is that the study represents a very modest increase in knowledge about N-glycanase’s role in regulating cellular metabolism and pathways involved in disease pathogenesis.

Summary – the manuscript presents a proteomic analysis of changes in protein levels in NGLY1 deficient HepG2 cells, noting a pronounced disturbance in pathways implicated in iron metabolism and ferroptosis, an iron-dependent oxidative form of cell death. The authors characterize changes in protein and mRNA levels associated with iron handling and ferroptosis, observe elevated iron influx, changes in key transferrin pathway members, increases in reactive oxygen species and lipid peroxidation.

Criticisms:
The manuscript details changes in markers/proteins/genes associated with ferroptosis regulation, but does not directly measure whether NGLY1 loss in HepG2 cells affects sensitivity to induction of ferroptosis (a form of cell death) as was reported earlier by Forcina et al (2022) <https://doi.org/10.1073/pnas.2118646119>. As result, statements such as “ferroptosis is prominently upregulated” (abstract), cells are in a “perpetual state of ferroptosis” (p. 4, 14) are misleading. More accurate would be to state that pathways implicated in ferroptosis regulation are altered. This is addressed somewhat in the discussion section, in which the authors note that the mutant cells are capable of cell growth and division, but may show “a ‘sublethal’ engagement of ferroptosis”. A better way to resolve this would be to measure whether the cells are in fact more sensitive to ferroptosis induction by, for example, GPX4 inhibitors like RSL3 or ML 162 as was shown by Forcina et al.
Following the reviewer’s suggestion, we examined the sensitivity of WT and *NGLY1*-KO HepG2 cells to GPX4 inhibitors, RSL3 and ML162. As a result, we found that *NGLY1*-KO HepG2 cells were actually more resistant to the induced ferroptotic cell death than WT HepG2 cells. As outlined above in more detail, our additional experiments confirmed the higher GPX4 expression in the *NGLY1*-KO HepG2 cells compared to WT HepG2 cells. Therefore, in the case of HepG2 cells, *NGLY1* deletion causes more GPX4 expression, which renders *NGLY1*-KO HepG2 more resistant to ferroptosis. These results were added as Fig. 4 in the revised manuscript.

The work is entirely conducted using a single cell line HepG2. (The section title in line 264 “NGLY1 deletion increased iron content in human liver cells” should refer to a “human liver cell line” rather than “human liver cells”). The findings would be substantially extended if similar changes were observed in primary liver cells, for example from NGLY1 deficient rodent models. Given that the data is based on mutant clones of an immortal cell line selected for the ability to grow (and thus has acquired compensatory mechanisms to survive increased ferroptotic/oxidative stress), there is reason to be concerned that phenotypic changes may be very different in primary cells.
This reviewer’s point is well taken. Unfortunately, however, we were not able to conduct experiments using primary cells in this study. To provide a broader cellular background, we performed additional experiments using *NGLY1*/*Ngly1*-KO and WT HEK293T and MEF cells. As included in the revised manuscript, the ferroptosis-related phenotypes appeared to be totally context-dependent (*i.e.*, HEK293T cells showed a similar trend to HegG2 cells, while MEF cells showed the opposite). The results of the additional experiments have been added to Supplemental Fig. 1. A similar context-dependency with respect to GPX4 mRNA and protein expression levels in NGLY1 deficiency patient-derived fibroblasts and lymphoblastoid cells has been reported in the “NGLY1 browser” (https://apps.embl.de/ngly1browser/) public database.

Minor criticisms:
- There are substantial differences in the levels of ROS generation between KO lines 1 and 2 (Figure 3D). Is there any explanation for that difference? Nonetheless, the authors should be encouraged to note that difference, even if it does not change the conclusion of that analysis.

Following the reviewer’s recommendation, we mention the difference between clones in the revised manuscript. To this end, the sentence “Quantitative ROS assays further confirmed significantly elevated ROS levels in *NGLY1*-KO compared to WT HepG2 cells.” was revised to “Quantitative ROS assays further confirmed significantly elevated ROS levels in *NGLY1*-KO compared to WT HepG2 cells, while clonal differences in the degree of elevation were noted.” (page 11, lines 269-270).

At this time, we are unable to provide a clear explanation for the reasons behind the differences observed between clones.

- The figure legend to Figure 3E should provide an explanation for the yellow arrows (currently in the results section text)

Following the reviewer’s recommendation, we added the explanation to the figure legend (in the revised manuscript, Fig. 3E has been moved to Fig. 2G).

- Line 332: “believed to be the consequence of its perineal degradation” I believe the authors mean to say “persistent”, “constant”, or “constitutive” degradation.

The description was changed from “perineal” to “constitutive” (page 15, line 374).
