# Supplementary material for: Comparative proteomics of HepG2 cells reveals NGLY1 as an important regulator of ferroptosis resistance and iron uptake
Source: PLoS One. 2025 Aug 14;20(8):e0328166. doi: 10.1371/journal.pone.0328166 (PMC12352660; doi:10.1371/journal.pone.0328166)
Supplement: Attachment — Submitted filename: Response-to-reviewers-comments-2.docx [file pone.0328166.s005.docx]

**Response to reviewer’s comments:**

Reviewer #2:

We wish to thank this reviewer for the valuable comments that are intended to improve our manuscripts.

Comments:

Response to revised submission:
The authors have conducted additional studies and have responded substantially to reviewers’ comments and criticisms.

Summary – the manuscript presents a proteomic analysis of changes in protein levels in NGLY1 deficient HepG2 cells, noting a pronounced disturbance in pathways implicated in iron metabolism and ferroptosis, an iron-dependent oxidative form of cell death. The authors characterize changes in protein and mRNA levels associated with iron handling and ferroptosis, observe elevated iron influx, changes in key transferrin pathway members, increases in reactive oxygen species and lipid peroxidation.

In my earlier review I noted that the manuscript details changes in markers/proteins/genes associated with ferroptosis regulation, but did not directly measure whether NGLY1 loss in HepG2 cells affects sensitivity to induction of ferroptosis. In response to this criticism, the authors examined the ferroptosis sensitivity of wt and NGLY1 deficient HepG2 cells and found that in this cellular context, NGLY1 deficient cells are in fact more resistant to ferroptosis induced by GPX4 inhibition compared to wild type cells. This results differs from an earlier study (Forcina et al (2022) <https://doi.org/10.1073/pnas.2118646119>) with a different cell type which found that NGLY1 deficient cells re more sensitive to ferroptosis induction. The authors also conducted additional studies and found that GPX4 protein and mRNA levels are increased in NGLY1 deficient HepG2 cells, which can account for their increased resistance to ferroptosis inducers, and likely represents a compensatory mechanism to allow cellular survival in the face of increased oxidative stress. The authors also assessed ferroptosis sensitivity in other cell types and examined publicly available datasets. They found that in some cellular contexts, NGLY1 loss is accompanied by increased GPX4 expression and resistance to ferroptosis induction, while in other contexts, NGLY1 loss does not lead to increased GPX4 levels, and NGLY1 deficient cells are more sensitive to ferroptosis induction. Overall, these data indicate that NGLY1 loss leads to disturbances in iron metabolism and oxidative stress, and that some cell types are able to develop compensatory mechanisms to tolerate oxidative stress without undergoing ferroptosis.

With these revisions, the manuscript provides a more through and detailed analysis of the consequences of NGLY1 loss on iron homeostasis, oxidative stress, and ferroptosis, and publication is supported.

Two revisions are suggested:

First, as noted in my earlier review, statements such as “ferroptosis is prominently upregulated” (abstract, line 31), and cells are in a “perpetual state of ferroptosis” (p. 4, lines 81-82; p. 15 line 379) are misleading. In fact, as shown here, NGLY1 deficient cells are MORE RESISTANT to ferroptosis induction by GPX4 inhibition. Thus, a much better statement would be to propose that NGLY1 cells are in a state of increased oxidative “pro-ferroptotic” stress and are able to tolerate this state of increased stress by compensatory upregulation of GPX4.

Second the data presented in Supplemental figure 1, showing that NGLY1 inactivation in different cell types has varying effects on ferroptosis sensitivity and correlates with varying changes in GPX4 expression, should be included in the results section rather than the discussion section.

In accordance with this reviewer’s suggestions, we have changed the description as below.

**Revision to Figures**

1. Previous Figs. S1A-F were moved to Figs. 4E-J.

2. Previous Figs. S2A-C were moved to Figs. S1A-C.

*In accordance with these revisions, associated Figure references in the text and associated Figure legends were revised (pages 29-30, lines 688, 691-696, 713-714).

**Revision to Abstract**

1. page 2, lines 31-32: The sentence was changed from “quantities in *NGLY1*-deficient HepG2 cells that suggested ferroptosis was prominently upregulated” to “quantities in *NGLY1*-deficient HepG2 cells, indicating that these cells are under “pro-ferroptotic” stress state”.

**Revision to Introduction**

1. page 4, lines 82-83: The sentence was changed from “*NGLY1*-KO HepG2 cells were found to be perpetually in a state of ferroptosis, a regulated form of cell death characterized by the iron-dependent accumulation of lipid peroxides” to “*NGLY1*-KO HepG2 cells were found to be in a state of pro-ferroptosis stress”.

2. page 4, lines 77-79: Due to the above change, the following sentence was added: Ferroptosis is a regulated form of cell death characterized by the iron-dependent accumulation of lipid peroxides (7, 11, 12).

**Revision to Materials and Methods**

Due to changes in the Figs, the contents previously described in the Supplemental methods section have been added to the Materials and Methods section.

1. Culture methods for HEK293T cells and MEF cells were added to “Cell Culture and Treatment” section (page 5, lines 104-106, 108).

1. Culture methods for HEK293T cells and MEF cells were added to “Cell Culture and Treatment” section (page 5, lines 104-106, 108).

2. The *NGLY1*-KO method in HEK293T cells was added to “Knockout of *NGLY1* in HepG2 Cells” section (page 5, lines 109, 113, 117-118).

3. The ferroptosis inducer treatment method of HEK293T and MEF cells were added to “Ferroptosis inducer treatment of HepG2 cells” section (page 9, lines 211-214).

**Revision to Results**

1. The following sentences were added: Earlier reports indicated the heightened sensitivity to ferroptotic cell death of *NGLY1*-KO A549 cells as well as their diminished GPX4 protein levels (7). Therefore, we conducted the same experiments using cell lines other than HepG2 cells to examine the context-dependency of the impact of *NGLY1* loss on ferroptotic susceptibility. Our results, obtained by using HEK293T and MEF cells, showed that *NGLY1*-KO HEK293T cells exhibit increased resistance to induced ferroptotic cell death, accompanied by elevated GPX4 protein levels when compared with WT HEK293T cells (**Figs. 4 E, G, I**). Conversely, *Ngly1*-KO MEF cells exhibited heightened sensitivity to induced ferroptotic cell death, characterized by reduced GPX4 protein levels in comparison to WT MEF cells (**Figs. 4F, H, J**). Taken together, these results highlight the context dependency of induced ferroptotic cell death resistance and GPX4 expression levels on *NGLY1*-KO/*Ngly1*-KO cell type. (pages 12-13, lines 306-315)

**Revision to Discussion**

1. page 15, lines 376-381: The text passage “Earlier reports indicated the heightened sensitivity to ferroptotic cell death of *NGLY1*-KO A549 cells as well as their diminished GPX4 protein levels (7). The public database “NGLY1 browser (https://apps.embl.de/ngly1browser/)” reports that NGLY1 deficiency patient-derived fibroblasts show increased GPX4 mRNA and reduced GPX4 protein levels, while patient-derived lymphoblastoid cells showed decreased GPX4 mRNA and protein levels. Moreover, preliminary results indicate that *NGLY1*-KO HEK293T cells exhibit increased resistance to induced ferroptotic cell death, accompanied by elevated GPX4 protein levels when compared with WT HEK293T cells. Conversely, *Ngly1*-KO MEF cells exhibited heightened sensitivity to induced ferroptotic cell death, characterized by reduced GPX4 protein levels in comparison to WT MEF cells (**Figs. S1A-F**). Taken together, these results highlight the context dependency of induced ferroptotic cell death resistance and GPX4 mRNA and protein expression levels on *NGLY1*-KO cell type.” was revised to “Regarding the context dependency of GPX4 expression levels on *NGLY1*-KO cell type, the public database “NGLY1 browser (https://apps.embl.de/ngly1browser/)” reports that NGLY1 deficiency patient-derived fibroblasts show increased GPX4 mRNA and reduced GPX4 protein levels, while patient-derived lymphoblastoid cells showed decreased GPX4 mRNA and protein levels. This observation also highlights the context dependency of GPX4 mRNA and protein expression levels on *NGLY1*-KO cell type.”.

2. page 16, lines 389-390: “perpetual state of ferroptosis” was changed to “increased oxidative “pro-ferroptotic” state and the compensatory upregulation of GPX4 to tolerate this state”.
